# Supplementary material for: IMPELLA® or Extracorporeal Membrane Oxygenation for Left Ventricular Dominant Refractory Cardiogenic Shock
Source: J Clin Med. 2021 Feb 14;10(4):759. doi: 10.3390/jcm10040759 (PMC7918655; doi:10.3390/jcm10040759)
Supplement: Supplementary file 1 [file jcm-10-00759-s001.pdf]

Supplementary Materials

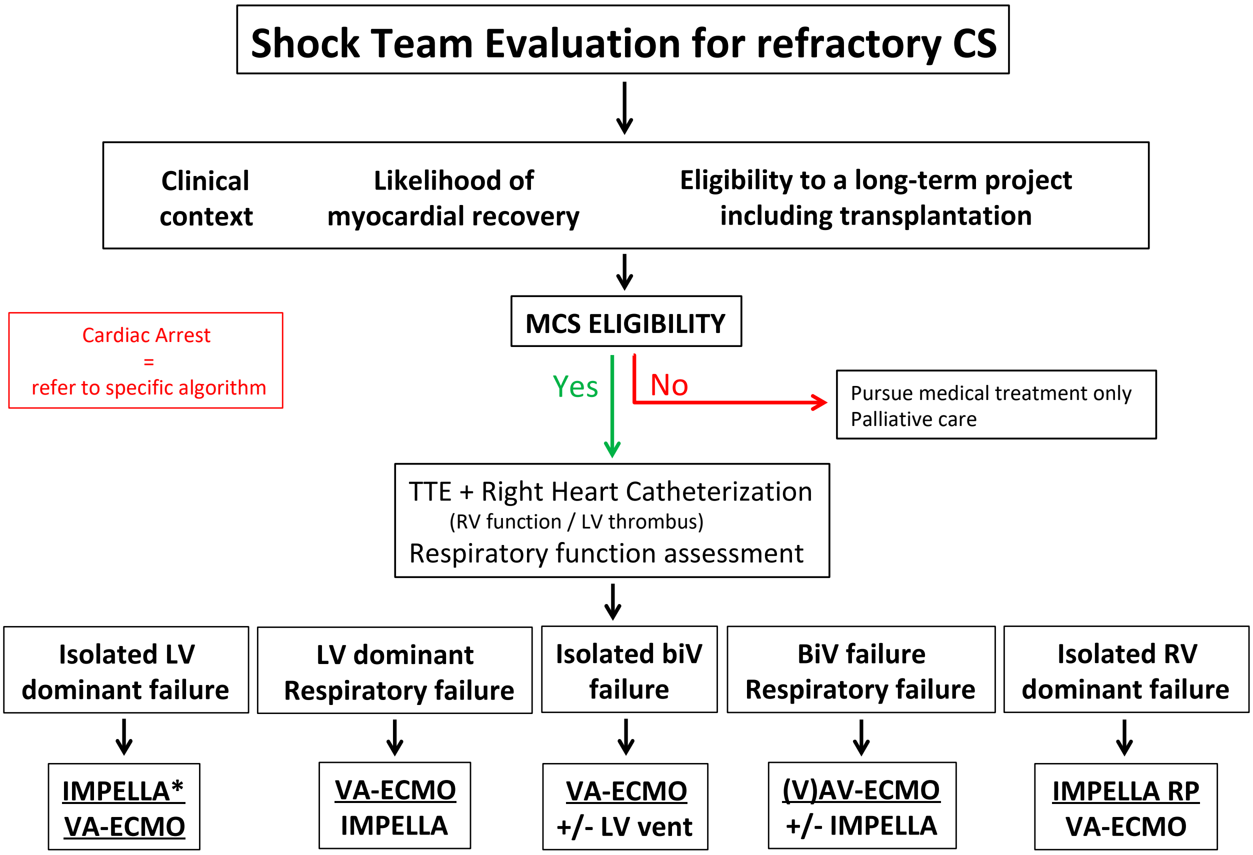

Figure S1. Local protocol for refractory CS management.

CS=cardiogenic shock; MCS=mechanical circulatory support; TTE=transthoracic echocardiography; LV=left ventricle; RV=right ventricle; biV=biventricular; VA-ECMO=veno-arterial extracorporeal membrane oxygenation; *Preferred strategy if AMI / pulmonary edema / moderate organ failure / acceptable RV function / LV thrombus absence

Table S1. Variables associated with 6-month mortality after adjustment (univariate analysis).

	**Total population** *n* **= 128**	**Alive at 6 Months** *n* **= 61**	**Dead at 6 Months** *n* **= 67**	***p***
Demographics and Medical History				
Age (years)	53.8 +/- 13.1	50.3 +/- 13.9	57+/- 11.5	0.003
Sex (male), n (%)	93 (72.7%)	38 (62.3%)	55 (82.1%)	0.012
Body mass index (Kg/m^2)	27 +/- 4.9	26.5 +/- 4.7	27.4 +/- 5	0.264
Diabetes, *n* (%)	25 (19.5%)	8 (13.1%)	17 (25.4%)	0.067
History of coronary artery disease, *n* (%)	34 (26.6%)	14 (23%)	20 (29.9%)	0.377
History of stroke, *n* (%)	4 (3.1%)	0	4 (6%)	0.120
Peripheral artery disease, *n* (%)	3 (2.3%)	0	3 (4.5%)	0.244
Renal failure before admission, *n* (%)	7 (5.5%)	3 (4.9%)	4 (6%)	0.999
Admission				
Acute myocardial infarction, n (%)	72 (56.3%)	31 (50.8%)	41 (61.2%)	0.237
LVEF (%)	21.8 +/- 15.5	21.4 +/- 14.1	22.1+/- 17	0.843
Creatinine (mg/L)	18 +/- 10.1	15.3 +/- 7.1	20.6 +/- 11.8	0.003
Hemoglobin (g/dL)	11.9 +/- 2.6	11.6 +/- 2.7	12.1 +/- 2.5	0.296
Lactate (mmol/L)	5.82 +/- 4.93	4.76 +/- 3.43	6.89 +/- 5.91	0.019
ASAT (IU/L)	460 +/- 749	455.7 +/- 904.5	464.3 +/- 555.9	0.950
ALAT (IU/L)	269.5 +/- 552.1	228.6 +/- 509.8	307.7 +/- 590.4	0.432
PT (%)	63.2 +/- 31.2	67.1 +/- 38.6	59.2 +/- 20.9	0.184
Bilirubin (mg/L)	13.2 +/- 14.5	12 +/- 12.3	14.4 +/- 16.5	0.405
CRP-us (mg/L)	68.7 +/- 75.9	60.1 +/- 68.7	78 +/- 82.8	0.236
Mechanical Support				
VA-ECMO, *n* (%)	107 (83.6%)	51 (83.6%)	56 (83.6%)	0.997
IMPELLA®, *n* (%)	41 (32%)	14 (23%)	27 (40.3%)	0.036
IMPELLA-CP®, *n* (%)	34 (26.6%)	13 (21.3%)	21 (31.3%)	0.224
VA-ECMO as first device, *n* (%)	97 (75.8%)	50 (82%)	47 (70.1%)	0.119
IMPELLA® as first device, *n* (%)	31 (24.2%)	11 (18%)	20 (29.9%)	0.119
Duration of MCS (Days)	8.6 +/- 9.2	9.1 +/- 11.1	8.1 +/- 7.2	0.544

Table S2. Variables associated with 6-month mortality after adjustment (Cox multivariate analysis, model 1).

	**Hazard Ratio**	**95% Confidence Interval**	***p***
Age (per year)	1.04	1.01-1.07	0.028
Sex male	4.90	1.87-12.89	0.001
Diabetes	1.54	0.74-3.22	0.249
Acute myocardial infarction at admission	2.19	1.11-4.32	0.025
LVEF (per %)	0.99	0.97-1.01	0.233
Lactate level (per one unit)	1.17	1.10-1.24	<0.001
Creatinine level (per one unit)	1.05	1.01-1.08	0.005
Hemoglobin level (per one unit)	0.94	0.83-1.06	0.324
VA-ECMO first	0.27	0.11-0.64	0.001

Cox model: age, sex, diabetes, acute myocardial infarction at admission, left ventricle ejection fraction, lactate level, creatinine level, hemoglobin level, VA-ECMO first. Variables of interest were selected based on the previous literature. LVEF = left ventricular ejection fraction; VA-ECMO = veno-arterial membrane oxygenation.

Table S3. Variables associated with 30-day mortality after adjustment (Cox multivariate analysis, model 2).

	**Hazard Ratio**	**95% Confidence Interval**	***p***
Age (per year)	1.04	0.99-1.07	0.059
Sex male	4.73	1.63-13.73	0.004
Acute myocardial infarction at admission	3.22	1.44-7.16	0.004
Lactate level (per one unit)	1.16	1.09-1.23	<0.001
Creatinine level (per one unit)	1.05	1.01-1.10	0.012
VA-ECMO first	0.38	0.18-0.74	0.007

Cox model: variables associated with 30-day mortality in univariate analysis (Cf. Table 3). VA-ECMO = veno-arterial membrane oxygenation.

Table S4. Variables associated with 6-month mortality after adjustment (Cox multivariate analysis, model 2).

	**Hazard Ratio**	**95% Confidence Interval**	***p***
Age (per year)	1.04	1.01-1.07	0.011
Sex male	3.43	1.59-7.41	0.002
Lactate level (per one unit)	1.16	1.10-1.22	<0.001
Creatinine level (per one unit)	1.04	1.01-1.07	0.003
VA-ECMO first	0.34	0.17-0.67	0.002

Cox model: variables associated with 6-month mortality in univariate analysis (Cf. Supplemental Table S1). VA-ECMO = veno-arterial membrane oxygenation.
